# Evaluating a virtual reality visual fields analyzer in an urban, underserved glaucoma & glaucoma suspect patient populations to identify disparities

**DOI:** 10.1007/s00417-025-06886-7

**Published:** 2025-07-07

**Authors:** Owais M. Aftab, Yash S. Shah, Anup Dupaguntla, Tracy Andrews, Rashika Verma, Jasmine Mahajan, Priya Tailor, Rita Vought, Bernard C. Szirth, Albert S. Khouri, Miriam M. Habiel

**Affiliations:** 1 Rutgers New Jersey Medical School, Institute of Ophthalmology and Visual Science, Doctor’s Office Center, 90 Bergen Street, Suite 6100, Newark, NJ 07103 USA; 2 Department of Ophthalmology, New York Eye and Ear Infirmary of Mount Sinai, Icahn School of Medicine at Mount Sinai, New York, NY 10029 USA; 3 Department of Biostatistics and Epidemiology, Rutgers School of Public Health, Piscataway, NJ USA; 4 Department of Ophthalmology, SUNY Downstate Medical Center, Brooklyn, NY USA; 5 Department of Ophthalmology, Medstar Georgetown Hospital,, Washington, DC USA

**Keywords:** Glaucoma, Portable perimetry, Social disparities

## Abstract

**Purpose:**

To evaluate the utility of wearable visual field perimetry in an urban, underserved patient population and identify disparities in its utility as a screening tool.

**Methods:**

175 eyes from 105 participants (46 non-glaucomatous eyes from 34 participants and 113 glaucomatous eyes from 74 participants; 16 eyes failed inclusion criteria) presenting at University Hospital in Newark, New Jersey for glaucoma evaluation underwent testing by both the Humphrey Visual Field Analyzer™ (HFA) and PalmScan VF2000 G2™. Glaucoma severity was classified as per the Hoddap criteria. Mean deviation (MD), pattern standard deviation (PSD), visual field index (VFI), mean sensitivity (MS), & area under the receiver operating curve (AUC) on analysis adjusted for inter-eye correlation.

**Results:**

The VF2000 and HFA significantly differed in VFI as the VF2000 consistently underestimated VFI (*p* = 0.003) but did not significantly differ in MD (*p* = 0.664) or PSD (*p* = 0.584). The VF2000 had significantly fewer false positives (*p* < 0.001) and fixation losses (*p* = 0.001) but was a significantly longer exam (*p* = 0.018). On a multivariate logistic regression model adjusting for both inter-eye correlation and demographic variables, the VF2000 had an AUC of 0.7007, indicating fair agreement when identifying severe glaucoma. Language, age, and sex did not independently impact odds of agreement between the two devices; however, differences based on the interaction of age and language were observed.

**Conclusion:**

Our analysis of the Humphrey Visual Field against the virtual reality PalmScan VF2000 G2™ in an urban, diverse population found subtle disparities in predictive staging of glaucoma. Future studies may need to account for these disparities by evaluating the combinations of demographic interactions rather than evaluating them as independent, unrelated factors.

**Key messages:**

***What is known***

- Portable perimetry and virtual reality headsets have been used with moderate efficacy in screenings for glaucoma, but gaps exist in the quality of results as compared to the Humphrey Visual Fields Analyzer.

***What is new***

- The PalmScan VF2000 G2, a portable perimetry headset, may be suitable as a screening device, but it is not advanced enough to differentiate glaucoma stage as effectively as HFA analysis.
- Disparities along social determinants of health do exist in VF2000 detection of glaucoma, though these manifestations may be subtle and tied to the interaction of many complex factors.
- Future studies may benefit from examining the interaction between demographic factors as variables predictive of outcome.

## Introduction

Glaucoma is a complex, multi-factorial disease frequently characterized by elevated intraocular pressure leading to visual field loss, and as such, it is a leading cause of irreversible blindness with over 70 million people affected worldwide [1]. Within the United States, glaucoma is known to be twice as high in African Americans as compared to that of Caucasians, with a prevalence of 3.4% in African Americans and 1.7% in Caucasians [2]. While this disparity may have some relationship with genetics and environmental factors, many cases of glaucoma within the African American population are not detected until the latter stage of disease [3, 4]. This may be due to limited access to medical education, community screenings, and intervention. Previous studies have shown that the disparities in eye care and in medical intervention between African American and Caucasian population persists, even after stratification for socioeconomic status [5]. The prevalence of undiagnosed glaucoma in African Americans between the ages of 50–59 is estimated to be 50%, though it is theorized that this rate could decrease to as low as 27% with implementations of nationwide glaucoma screenings [6]. Because of this, early access and treatment of glaucoma is essential, especially within at-risk populations.

Static perimetry with devices like the Humphrey Field Analyzer (HFA) is currently the gold standard method to test for visual field defects in glaucoma [7]. However, its use in community screenings is limited due to its heavy weight, limited transportability, and large footprint [8, 9]. A previous study exploring the use of portable technology as an alternative in detecting glaucoma suggested advantages in affordability, practicality, and the limited need for specialized training, especially in lower socioeconomic status areas where may be limited access to glaucoma care, HFA, and non-portable perimetry [10]. The PalmScan VF2000 G2™ visual field analyzer is a battery powered, wireless portable visual field device. It is relatively inexpensive in comparison to the HFA and designed to be easy to use. The machine can be programmed to give instructions in different languages and provides life feedback to patients during testing. The use of the VF2000 and other VR-based technologies, ranging from tablet-based perimetry to other custom-designed headsets, have been tested several times with respect to their accuracy in detecting glaucoma when using the HFA as a baseline, and they have generally been shown to provide moderate correlation in the detection and classification of glaucoma [11–14].

While the VF2000 has been evaluated internationally, formal evaluation for its efficacy in underserved, urban populations in the US have been limited. Given the ease of use and language translation features, we sought to confirm the efficacy of the VF2000 as a screening device for glaucoma in diverse, lower socioeconomic status populations and to compare it with standard HFA testing in this population in Newark, New Jersey.

## Methods

This study was a single-center cross-sectional observational analysis of 175 eyes collected from 105 participants to compare portable VFA testing from the PalmScan VF2000 G2 ^TM^ to results from the Humphrey Visual Field Analyzer (HFA). This study was performed at the Rutgers New Jersey Medical School, a tertiary care center located in Newark, New Jersey, and was approved by the medical school’s Institutional Review Board. All participants were recruited for participation in this study after obtaining informed consent as per the tenets of the Declaration of Helsinki.

Participants were recruited from an established academic practice glaucoma clinic, which is primarily composed of patients receiving charity care, patients that have limited health literacy, and/or patients that have several socioeconomic as well as systemic barriers to ophthalmologic care. Participants were diagnosed and stratified into glaucomatous and non-glaucomatous groups by one of two glaucoma specialists. The diagnostic criteria for glaucoma was based on the American Academy of Ophthalmology’s Preferred Practice pattern for each subset of glaucoma and included one or more of the following: characteristic optic nerve changes on clinical exam and/or on ocular Coherence Tomography (OCT testing) such as notching, retinal nerve fiber layer (RNFL) defects or macular ganglion cell loss, and/or visual field defects consistent with RNFL damage [15]. Glaucomatous patients were further stratified by severity as per HFA results as per the Hoddap criteria [16].

Diagnostic criteria of glaucoma suspects was based on the Ocular Hypertensive Treatment trial and took account risk factors such as ethnicity, family history, central corneal thickness, vertical cup to disk ratio and intraocular pressures along with PSD on HVF testing [17]. Inclusion criteria included patient age of 18 years or older; patients with visual acuity < 20/200 or had any other comorbidities likely to confound data such as corneal or macular pathologies were excluded, in line with previous studies [11]. Data collected from patients with an unreliable HFA test were excluded (see below). Further, patients with non-glaucomatous illnesses that may affect the visual field and patients with other illnesses like neurological or psychiatric disorders that prevented examination were excluded. All participants were provided informed consent in English or a language of their choosing using an interpreter service as needed.

All participants’ eyes were tested using HFA and the VF2000 on the same day, with an interval of an at least ten-minutes between tests. Participants were randomly assigned to have HFA first or VF2000 first. 45 eyes underwent VF2000 testing followed by HFA testing, while 74 underwent HFA testing first.

### Testing procedure

For HFA visual field analysis, participants were seated in a dark room and had their untested eye covered with an eyepatch. Refractive error was corrected. Patients were instructed to focus on a central fixation light and to press a clicker whenever they saw a light flash. The Swedish Interactive Thresholding Algorithm (SITA) Standard 24 − 2 was used in all eyes. Glaucomatous eyes were further subclassified into mild (>−6 dB), moderate (−6 through − 12 dB), or severe (<−12 dB) glaucoma based on HFA results as per the aforementioned Hoddap Classification. Participants with a false positive (FP) or false negative (FN) rate > 33% on HFA were excluded from the study in accordance with past literature [18]. Fixation loss criteria were not utilized in the exclusion criteria in accordance with past evaluations of portable perimetry [18], because the fixation loss cutoffs have both been relaxed [19] and had their validity questioned [20] in the past.

**Fig. 1:**
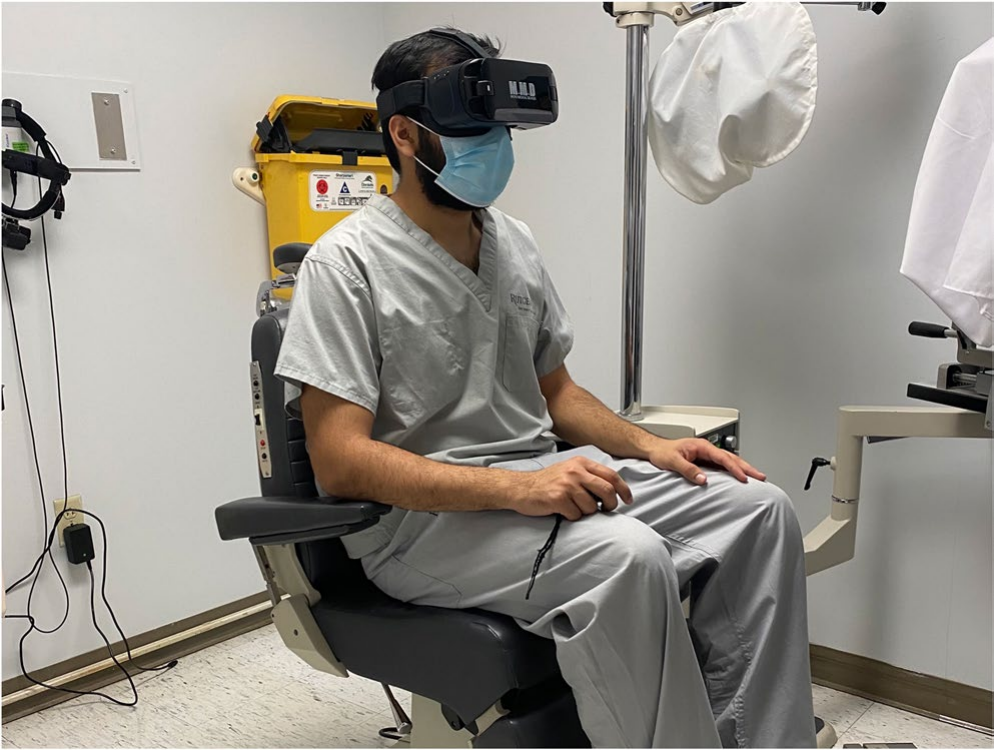
Example an individual wearing the VF2000 portable perimetry device

For the PalmScan VF2000 visual field analysis, participants were seated in a dimly lit room. All patients were new to any form of virtual reality visual field testing. The virtual reality goggles were placed on the subject and straps were adjusted for comfort (Fig. 1). Patients were given instructive audio prompts from the VF2000 unit in their native language (languages available included English, Spanish, Portuguese, French, Arabic, and Hindi, amongst others). Refractive error was adjusted for using the VF2000 unit’s focus wheel. No eyepatch was necessary as the mobile headset contains a built-in occluder. Participants were then instructed to focus on a central fixation light and press a clicker whenever a peripheral light was visualized. The central 24 − 2 test was performed on the VF2000 unit with a stimulus size of three, presentation time of 200 milliseconds, age-adjusted speed (with a baseline of 0.6 s), and background dB of 31.5 apostilb. Note that the Normative Database for the VF2000 system is based on data from 762 eyes from patients who have been examined and deemed to be without any ocular disease (e.g., glaucoma). Further, the screen is an LCD screen and there are no light diodes involved.

### Testing strategy

The testing strategy used for this study was ‘Full Threshold Fast’ and was designed distinctively for each age group. It is a variation of the ‘Full Threshold from Master’ strategy whereby an average is taken for the test amongst a particular age group and each test thereafter is calibrated against the average. During the test, a point is highlighted at 2 decibels higher than the average, and if the patient cannot see it, it is shown again. If missed again, this point is bracketed, i.e., the point is highlighted with a range of intensities, and the intensity at which the patient can see it is marked on the test. The principle behind the ‘Full Threshold Fast’ strategy is that 4 adjacent points, one in the center of each quadrant, are chosen. Each point is bracketed, and a value of intensity is assigned to each point. All the points around the center point are then calibrated against that center point instead of testing each point individually, which theoretically allows for faster testing.

For each HFA test, mean standard deviation (MD), pattern standard deviation (PSD), and visual field index were recorded in addition to false positives, false negatives, and fixation losses and similarly, for VF2000 tests, MD, PSD, VFI, and mean sensitivity (MS) were recorded in addition to false positives, negatives, and fixation loses.

### Statistical analysis

To determine the number of patients to recruit for the study, we performed a sample size calculation utilizing a test for one ROC curve with an alpha of 0.050, target power of 0.80, assumption of AUCROC of the disadvantaged cohort of 0.700 and assumption of an AUCROC of 0.9750 in non-disadvantaged populations, in-line with prior studies evaluating the AUCROC of portable perimetry in both disadvantaged [10] and non-disadvantaged [21] populations. A minimum sample of 18 patients was determined necessary by the sample size calculation.

Test parameters collected from the HFA and VF2000 were first evaluated for agreement and variation followed using statistical analyses in accordance with past literature [11, 18]. Variables were first assessed for normality using the Kolmogorov-Smirnov test. As all variables were non-parametric, demographic variability between the glaucomatous and non-glaucomatous cohort in addition to variation in HFA and VF2000 parameters across all eyes were compared using the Mann-Whitney U-test or Chi-Square test as indicated. A multivariable logistic regression model with main effects for age, language, and sex, and with an interaction for language by age tested agreement between HFA and VF2000 glaucoma staging. The model was adjusted for potential autocorrelation within subjects who had both eyes tested. Area Under the Curve Receiver Operator Curves were plotted to assess the efficacy of the VF2000 technology. An alpha level of *p* < 0.05 was used to evaluate significance, and statistical analyses were performed using SPSS Statistics Version 26.0 (IBM Corporation, Armonk, NY) as well as STATA Version 18.0 (StataCorp LLC, College Station, Texas).

All procedures performed in studies involving human participants were in accordance with the ethical standards of the institutional and/or national research committee and with the 1964 Helsinki Declaration and its later amendments or comparable ethical standards. The study was approved by the Institutional Review Board of Rutgers NJMS (IRB # Pro20140001070).

## Results

We surveyed a total of 172 eyes from 106 patients, we excluded 16 eyes from 10 patients due to either false positives or false negatives > 0.33 on their HFA test. 2 participants had agreed for testing but were unable to complete testing on either the HFA or the VF2000. A summary of variation in demographic information from across glaucomatous vs. non-glaucomatous cohorts is presented in Table 1. Non-glaucomatous and glaucomatous cohorts did not significantly vary in age, sex, or language. Of the total number of eyes, 46 (28.9%) eyes from 34 participants were non-glaucomatous but glaucoma suspects and 113 (71.1%) eyes from 74 participants were glaucomatous. Summary statistics for test parameters from all eyes are displayed in Table 2. While the median VFI found by the HFA was significantly higher than the VFI found by the VF2000 (*p* = 0.003), MD (*p* = 0.664) and PSD (*p* = 0.584) did not significantly differ between the two devices. Notably, the VF2000 had significantly fewer false positives (*p* < 0.001) and fixation losses (*p* = 0.001), though it had a significantly longer test duration (*p* = 0.018).

**Table 1:** Comparison of glaucoma vs. non-glaucoma cohorts

Variable	Glaucoma diagnosis		
	Eyes		
	Suspected	Confirmed	*P*-value
	*N* = 46	*N* = 113	
Ethnicity			0.075
Non-Hispanic Black	23 (50.00%)	50 (44.25%)	
Non-Hispanic White	7 (15.22%)	8 (7.08%)	
Hispanic	15 (32.61%)	55 (48.67%)	
Language			0.062
English	33 (71.74%)	63 (55.75%)	
Non-English	13 (28.26%)	50 (44.25%)	
Age Cohorts			0.079
Geriatric (65+)	19 (41.30%)	64 (56.64%)	
Non-Geriatric (18–65)	27 (58.70%)	49 (43.36%)	
14–54	13 (28.26)	22 (19.47%)	0.396
55–69	22 (47.83(	55 (48.67%)	
70+	11 (23.91)	36 (31.86%)	
Female Gender	20 (43.48%)	67 (59.29%)	0.069
HFA VFI	0.98 (0.95, 0.99)	0.67 (0.32, 0.88)	**< 0.001**
HFA MSD	−1.45 (−2.77, −0.26)	−13.63 (−22.64, −6.35)	**< 0.001**
HFA PSD	1.90 (1.59, 2.70)	6.41 (3.85, 9.17)	**< 0.001**
False positive on HFA	0.01 (0.00–0.06)	0.02 (0.00–0.05)	0.81
False positive on VF2000	0.00 (0.00–0.00)	0.00 (0.00–0.00)	0.16
False negative on HFA	0.03 (0.00–0.07)	0.07 (0.00–0.13)	**0.031**
False negative on VF2000	0.00 (0.00–0.00)	0.12 (0.00–0.38)	**< 0.001**

**Table 2:** Comparison of test parameters between HFA and VFA

Test parameter	HFA median (IQR)	VF2000 median (IQR)	*P*-value
VFI	0.86 (0.51, 0.96)	0.69 (0.36, 0.87)	**0.003**
MD	−7.58 (−17.69, −2.76)	−8.68 (−17.57, −3.43)	0.664
PSD	4.9 (2.47, 8.21)	5.32 (3.26, 7.90)	0.584
False positive	0.02 (0.00, 0.05)	0.00 (0.00, 0.00)	**< 0.001**
False negative	0.06 (0.00, 0.05)	0.13 (0.00, 0.25)	0.351
Fixation loss	0.18 (0.00, 0.1275)	0.10 (0.00, 0.375)	**0.001**
Time (seconds)	395.00 (327.00, 461.00)	428.00 (359.50, 487.50)	**0.018**

**Fig. 2:**
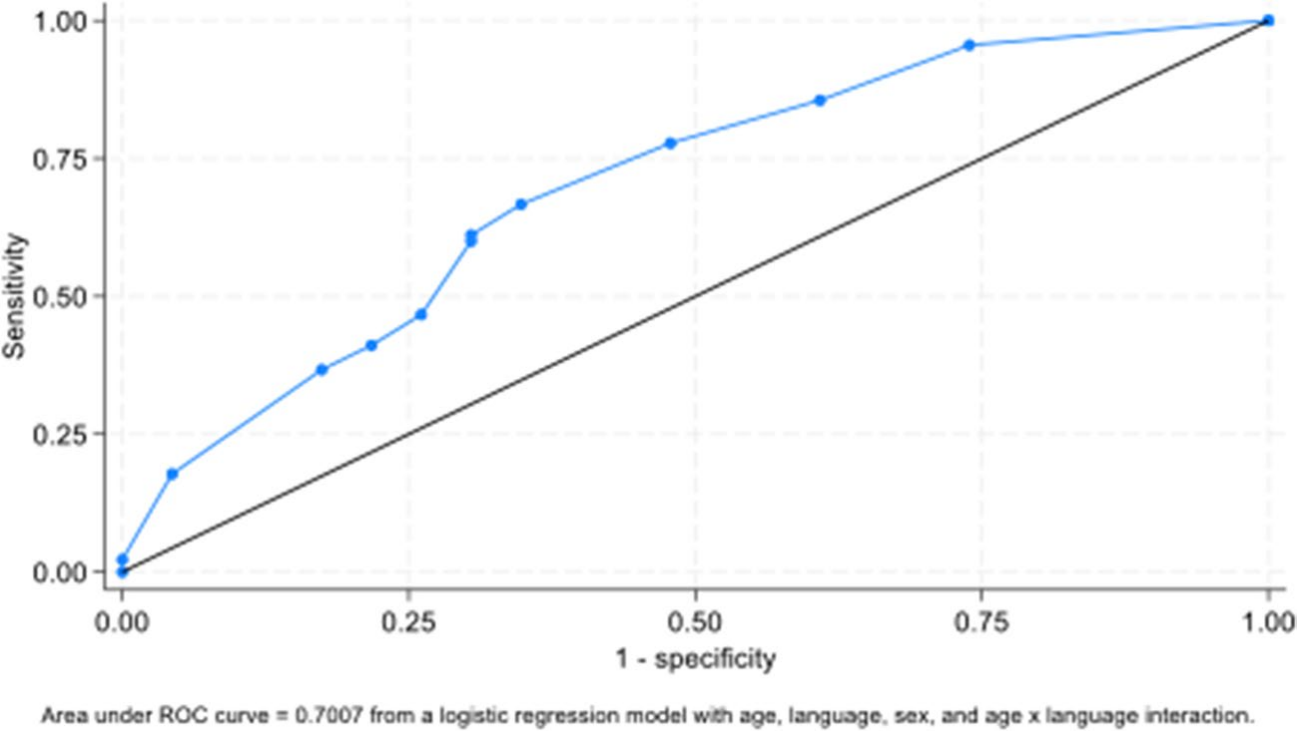
Receiver operator curve: agreement in determination of severe glaucoma – HFA vs. VR

Follow-up analysis was then performed to ascertain the overall agreement between the HFA and VF2000 in identifying severe glaucoma through a multivariate regression model with main effects for age, language, and sex, and with an interaction for language by age. An AUC of 0.7007 was found (Table 3; Fig. 2) when evaluating agreement between the VF2000 and the HFA in identifying severe glaucoma. AUC values of 0.7–0.8 are considered acceptable while 0.8–0.9 are considered excellent and 0.9–1.0 are considered outstanding [22].

**Table 3:** Odds of agreement between HFA and VF2000 on determination of severe glaucoma

Odds of agreement between HFA and VR on determination of severe glaucoma				
HFA-VR agreement (Severe vs. Not)	Odds ratio	95% confidence interval		*P*-value
Model estimates				
Main effects				
Language (ref = English Speaker)				
Non-English Speaker	4.73	0.50	44.52	0.175
Age (ref = 70+)				
14–54.9	1.12	0.18	7.00	0.906
55–69.9	1.66	0.36	7.67	0.514
Female	0.58	0.19	1.72	0.324
Language by age interaction (ref = 70+)				
Non-English Speaker: Age 14–54.9	0.15	0.01	3.55	0.240
Non-English Speaker: Age 55–69.9	0.04	0.00	0.61	**0.020**
Probability of agreement between HFA and VR in determination of severe glaucoma for language by age interaction				
English Speaker: Age 14–54.9	0.81	0.60	1.03	
English Speaker: Age 50–69.9	0.87	0.75	0.98	
English Speaker: Age 70+	0.80	0.62	0.98	
Non-English Speaker: Age 14–54.9	0.76	0.45	1.07	
Non-English Speaker: Age 55–69.9	0.57	**0.34**	**0.80**	
Non-English Speaker: Age 70+	0.95	0.85	1.04	
AUC	0.7007	0.576	0.826

**Fig. 3:**
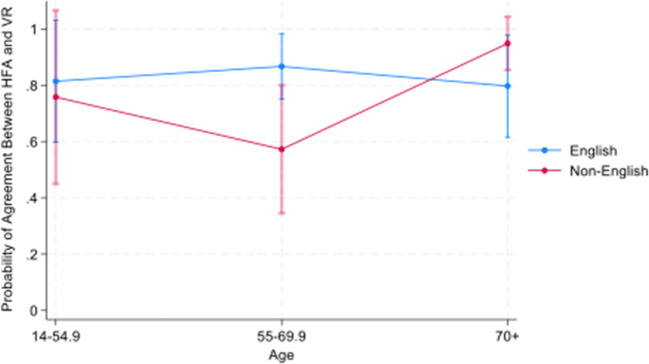
Probability of agreement in determination of severe glaucoma: HFA vs. VR

There were no independent effects of sex (*p* = 0.324), age (*p* = 0.906), or language (*p* = 0.175), However, there was a language by age interactions effect (Fig. 3). Non-English speakers age 55–69 were less likely to have agreement on their tests than Non-English speakers age 70+ (*p* = 0.02). Moreover, they were less likely than English speakers of the same age to have agreement on the two tests (*p* < 0.05).

## Discussion

We utilized the PalmScan VF2000 G2 in a clinically underserved, urban population from Newark, New Jersey with moderate success when evaluated against the static perimetry using the HFA as a gold standard to both identify glaucoma and stratify glaucoma severity. The VF2000 G2 generally yielded reliable results as the median false positives and false negatives were well-below our exclusion criteria of 33%, though we found that its median test time was longer than that of the HFA. This differs from past analyses which found portable perimetry was faster [11], though this could partly be explained by a significant portion of non-English speaking patients and geriatric patients, as it is well-known that these groups are uniquely susceptible to challenges in technological and digital literacy [23]. Regardless, the VF2000 was efficacious in differentiating glaucomatous eyes from non-glaucomatous eyes, and within glaucomatous eyes, it was most efficacious in identifying severe disease as compared to mild/moderate disease.

A previous evaluation of the VF2000 in India from the Laxmi Eye Hospital, a tertiary care center, found perfect agreement between the HFA and VF2000 when differentiating glaucomatous vs. non-glaucomatous eyes, which differed from our finding of acceptable rather than perfect agreement on AUC analysis [11]. It is important to note our model utilized an adjusted multivariate regression to account for confounding variables such as inter-eye correlation and demographic variables, rather than directly evaluating raw data. Regardless, our finding of the VF2000’s limited ability compared to prior studies may be expected given the challenges and limited health literacy of our patient population [14]. Most notably, despite these challenges, we found an acceptable AUC. This presents the opportunity for use in rural and mobile health settings, thereby allowing underserved populations to be tested earlier and in larger numbers. While there is some compromise in terms of the discriminative ability of the diagnostic tool, its ease-of-use ability can make it an important supplement to communities that are not receiving these services.

To our knowledge, this is the first evaluation of the VF2000 in a clinically underserved, urban setting in the United States. It is well-known that socioeconomically disadvantaged patient populations similar to those of Newark present with clinically complex cases of glaucoma due to disparities in insurance eligibility, education, income, and technological access [24], and this complex patient population may partly explain why we found only fair to moderate agreement between the VF2000 and HFA. While past literature found that the VF2000 performed the strongest [11] in differentiating severe glaucoma from mild glaucoma or moderate glaucoma, our results indicate limitations in the strength of portable perimetry’s ability to identify severe glaucoma in this urban center after adjustment for inter-eye correlation and other demographic variables. and other portable perimetry devices [14, 18, 25–27]. This study further emphasizes that while current perimeter devices may hold potential in screening or home monitoring for glaucoma due to their ability to differentiate glaucomatous eyes from non-glaucomatous eyes, they likely are not sufficient to supplant evaluation by HFA or other relevant workup in clinic because gaps in staging of disease remain.

We also evaluated the VF2000’s classification of mild/moderate vs. severe glaucoma across demographic variables, and to our knowledge, this is the first study to do so for portable perimetry. Factors like ethnicity, age, and sex have been previously identified as risk factors in the development of glaucoma [24], which is why it is important that any novel screening or diagnostic tools are efficacious across all demographic subgroups for early detection and management. Previously, it has been found that ethnic disparities exist as African American glaucoma participants demonstrated larger visual field variability over time as compared to European patients [28]. Additionally, it has previously been described that as compared to those of European descent, African American patients were 32% less likely to have an annual eye examination [29] and that Medicare beneficiaries of Hispanic and Africa descent were less likely to undergo preventative testing, utilize eye care, and have ophthalmologic appointments [5, 28]. Given these potential disparities in care are compounded by ethnic variations in optic nerve anatomy [30], it is especially important to identify glaucomatous disease and its stage in patients from underserved ethnicities.

Language has historically been noted as a barrier in care due to complications in communication between the provider and patient [31], and this is especially relevant when utilizing technical testing in the clinic due to the need of an interpreter, which may serve as a barrier to follow-up [32]. Other pertinent demographic factors to consider include sex and age as both sex [33] and geriatric age [34] are risk factors for primary angle closure glaucoma and glaucoma, respectively. In this study, we find that the relationship between language and age has a significant effect on the odds of agreement between the two test modalities. Studies that do not account for age when considering the effect of ethnicity and/or language may be overstating the diagnostic reliability of the VF2000. This may be a notable finding as social disparities may subtly manifest as the interaction between multiple factors in line with the biopsychosocial model of health, requiring an even greater effort on the part of healthcare providers to successfully combat these social determinants of health.

Finally, it may be pertinent to note that variability observed between the VF2000 and the HFA may indicate challenges with reliability between diagnostic technologies in an under-resourced population. Two challenges that face this population include a lack of familiarity with complex ophthalmological testing in addition to atypical clinical presentations. It is well-established that patients of lower socioeconomic status, as is typical of the community in Newark, are more likely to be diagnosed with end-stage disease [24], though several other factors like systemic racism and insurance regulation may also be involved as differences in eyecare utilization and outcomes have been found to persist even after adjustment for ethnic variation [5]. Another reason our results may have differed from this previous studies in addition to ethnic variability in visual field testing [35] include ethnic variation in the structure of the optic nerve [30]. Further, we recruited from a an underserved patient population where many patients in our practice are new patients from the community that are not regularly exposed to ophthalmologic diagnostic testing. This may increase external validity as this sample would theoretically be more representative of the community than an established practice.

Despite this, it is important to note that some limitations of this study include its recruitment from a clinically underserved population. Complex presentations in addition to a lack of consistent follow-up and familiarity with ophthalmologic examination may have caused an underestimation of the efficacy of the VF2000, though it is important to note that this patient population may be more reflective of those observed in large-scale community screenings. Unfortunately, only glaucoma and glaucoma suspects were included in this study, and there is a lack of a non-glaucomatous control group containing patients without any pathological changes of the disc and the visual field due to workflow limitations. Further, 35.2% of patients were first-time users of HFA, while all were first-time users of VF2000; this could negatively affect the reported effectiveness of VF2000 due to a lack of correction of a learning effect. Additionally, only patients with reliable HFA tests were included in the study, which may result in an overestimation of the efficacy of the VF2000’s performance. Further, only one clinician’s impression was used as a reference to compare the HFA and VF2000 results, and variations in clinical classification, especially in more advanced cases or atypical presentations, could slightly alter repeatability of this study. Patients were also assigned to either perform the HFA first or the VF2000 first based on whatever was best to avoid disrupting the flow of the clinic, and a more robust randomization technique could have been utilized to further limit any confounding test-retest variation. Additionally, the VF2000 does not allow for cylindrical correction of refractive error, whereas the HFA allows for both sphere and cylinder. The absence of correction of astigmatism may have artificially decreased the efficacy of VF2000 testing in this study. Additionally, while the majority of patients were first-time users of both the HFA and VF2000, some had past experience with the HFA. Differences in learning curves alongside a poor understanding of the learning curve for VR headset perimetry is an additional limitation of this study. Further, the VF2000 G2 does not have eye tracking technology, though newer headsets like the VF2000 NEO do.

In summary, our study confirmed the finding that the VF2000 may be suitable as a screening device, though it is not advanced enough to differentiate glaucoma stage as effectively as HFA analysis. Further, our study suggests that disparities along social determinants of health do exist, though their manifestation may be subtle and tied to the interaction of many complex factors. Future studies may benefit from examining the interaction between demographic factors as variables predictive of outcome. Future studies could also build upon these results by evaluating portable perimetry in high-volume screening settings, in at-home monitoring of glaucoma progression, or as a remote telemedicine tool in situations without direct access to an ophthalmologist.
